# Gauging NOTCH1 Activation in Cancer Using Immunohistochemistry

**DOI:** 10.1371/journal.pone.0067306

**Published:** 2013-06-18

**Authors:** Michael J. Kluk, Todd Ashworth, Hongfang Wang, Birgit Knoechel, Emily F. Mason, Elizabeth A. Morgan, David Dorfman, Geraldine Pinkus, Oliver Weigert, Jason L. Hornick, Lucian R. Chirieac, Michelle Hirsch, David J. Oh, Andrew P. South, Irene M. Leigh, Celine Pourreyron, Andrew J. Cassidy, Daniel J. DeAngelo, David M. Weinstock, Ian E. Krop, Deborah Dillon, Jane E. Brock, Alexander J. F. Lazar, Myron Peto, Raymond J. Cho, Alexander Stoeck, Brian B. Haines, Sriram Sathayanrayanan, Scott Rodig, Jon C. Aster

**Affiliations:** 1 Department of Pathology, Brigham and Women’s Hospital and Harvard Medical School, Boston, Massachusetts, United States of America; 2 Departments of Pediatric Hematology/Oncology and Medical Oncology, Dana Farber Cancer Institute, Boston, Massachusetts, United States of America; 3 Division of Cancer Research and Genetics Core Services Unit, Medical Research Institute, Ninewells Hospital and Medical School, University of Dundee, Dundee, United Kingdom; 4 Department of Pathology, M. D. Anderson Cancer Center, Houston, Texas, United States of America; 5 Knight Cancer Institute, Oregon Health & Science University, Portland, Oregon, United States of America; 6 Department of Dermatology, University of California, San Francisco, San Francisco, California, United States of America; 7 Merck Oncology, Boston, Massachusetts, United States of America; University of Navarra, Center for Applied Medical Research, Spain

## Abstract

Fixed, paraffin-embedded (FPE) tissues are a potentially rich resource for studying the role of NOTCH1 in cancer and other pathologies, but tests that reliably detect activated NOTCH1 (NICD1) in FPE samples have been lacking. Here, we bridge this gap by developing an immunohistochemical (IHC) stain that detects a neoepitope created by the proteolytic cleavage event that activates NOTCH1. Following validation using xenografted cancers and normal tissues with known patterns of NOTCH1 activation, we applied this test to tumors linked to dysregulated Notch signaling by mutational studies. As expected, frequent NICD1 staining was observed in T lymphoblastic leukemia/lymphoma, a tumor in which activating *NOTCH1* mutations are common. However, when IHC was used to gauge NOTCH1 activation in other human cancers, several unexpected findings emerged. Among B cell tumors, NICD1 staining was much more frequent in chronic lymphocytic leukemia than would be predicted based on the frequency of *NOTCH1* mutations, while mantle cell lymphoma and diffuse large B cell lymphoma showed no evidence of NOTCH1 activation. NICD1 was also detected in 38% of peripheral T cell lymphomas. Of interest, NICD1 staining in chronic lymphocytic leukemia cells and in angioimmunoblastic lymphoma was consistently more pronounced in lymph nodes than in surrounding soft tissues, implicating factors in the nodal microenvironment in NOTCH1 activation in these diseases. Among carcinomas, diffuse strong NICD1 staining was observed in 3.8% of cases of triple negative breast cancer (3 of 78 tumors), but was absent from 151 non-small cell lung carcinomas and 147 ovarian carcinomas. Frequent staining of normal endothelium was also observed; in line with this observation, strong NICD1 staining was also seen in 77% of angiosarcomas. These findings complement insights from genomic sequencing studies and suggest that IHC staining is a valuable experimental tool that may be useful in selection of patients for clinical trials.

## Introduction

Notch receptors participate in a conserved signaling pathway that regulates many cellular phenotypes, including cell fate, cell proliferation, and cell survival (for review, see [1]). Mammals possess four Notch genes (*NOTCH1*-*4*), each with distinct patterns of expression and different knockout phenotypes in mice. Canonical signaling through all four receptors relies on a series of ligand-dependent proteolytic cleavages, the last of which is carried out by a multisubunit protease called gamma-secretase, which cleaves Notch receptors within the inner half the receptor’s transmembrane domain. This liberates the Notch intracellular domain, NICD, which translocates to the nucleus and forms a short-lived transcriptional activation complex.

Outcomes produced by Notch signaling in normal cells vary dramatically with cellular context, presumably because epigenetic patterning of genomes leads to varied effects of NICD on transcriptional outputs. Genotypic data from humans and experimental results from mice indicate that *NOTCH1* also has varied roles in cancer, acting as either an oncogene or a tumor suppressor gene depending on cellular context. Gain-of-function mutations of *NOTCH1* are common in T lymphoblastic leukemia/lymphoma (T-LL) [2,3], and have also been described in subsets of chronic lymphocytic leukemia (CLL) [4–6], mantle cell lymphoma (MCL) [7], diffuse large B cell lymphoma [8], peripheral T cell lymphoma (PTCL) [9], breast cancer [10], and non-small cell lung cancer (NSCLC) [11]. These activating mutations include diverse point substitutions, deletions, and translocations that produce ligand-independent NOTCH1 proteolysis and activation, as well as mutations that remove a C-terminal PEST degron domain and thereby stabilize NICD1. In addition, data emerging from deep sequencing of cancer genomes has identified frequent mutation of genes encoding Notch pathway components in high-grade ovarian serous carcinomas [12], although the functional consequences of these mutations on Notch signaling is uncertain. There is also evidence that NOTCH1 has important functional roles in endothelium and other stromal components that may contribute to the malignant behavior of cancers [13,14]. Conversely, loss-of-function mutations distributed over a large part of the *NOTCH1* locus are common in squamous cell carcinomas of the skin [15] and head and neck [16,17] and also occur in a smaller subset of squamous cell carcinomas of the lung [15,18]. Similarly, loss of *notch1* function in vascular endothelium leads to angiosarcoma-like proliferations in mice [19,20].

There is interest in therapeutic targeting of NOTCH1 in cancers in which it has an oncogenic role with antagonists such as inhibitory antibodies and gamma-secretase inhibitors (GSI) [21]. Ideally, such trials would focus on treatment of patients whose tumors show evidence of ongoing NOTCH1 activation. Given the large size of the *NOTCH1* locus and the diversity of genetic aberrations that produce ligand-independent activation of NOTCH1 or stabilize NICD1, genetic screening for oncogenic *NOTCH1* alterations is challenging, particularly when working with archival FPE samples. Moreover, it is suspected that ligand-mediated NOTCH1 activation also contributes to tumor cell growth and survival, and such tumors would go undetected by genetic screening.

An ideal biomarker test would detect NICD1 within tumor cells directly, regardless of the underlying mechanism of NOTCH1 activation. To this end, we developed a robust, specific immunohistochemical (IHC) staining method that detects NICD1 in archival samples. The test relies on a commercial rabbit monoclonal antibody, previously used only in Western blot analyses, that is specific for a neoepitope in NICD1 created by gamma-secretase-mediated proteolysis of NOTCH1, the event that triggers NOTCH1 signaling. Following optimization and validation of the test using cancer xenografts bearing diverse *NOTCH1* aberrations and normal tissues, we screened a large series of human cancers of unknown *NOTCH1* mutational status for activated NOTCH1. Most T-LLs and CLLs showed evidence of ongoing NOTCH1 activation, as did many peripheral T cell lymphomas and a small subset triple-negative breast cancers. Activation of NOTCH1 was also detected in a majority of angiosarcomas, in line with the observation that NICD1 is readily detectable in normal endothelial cells within tumor stroma. By contrast, little or no NOTCH1 activation was observed in mantle cell lymphoma, diffuse large B cell lymphoma, non-small cell lung cancers, and ovarian carcinoma. These findings indicate that NOTCH1 activation among B cell tumors is both more sharply restricted to and more prevalent in CLL than would be predicted from prior genotypic data, raise questions about the proposed tumor suppressive role for NOTCH1 in vascular neoplasms, and suggest that IHC testing for NICD1 can be used to select patients for clinical trials of Notch pathway inhibitors, particularly those involving patients with tumors such as triple-negative breast cancer, in which NOTCH1 activation is confined to a small subset of tumors.

## Materials and Methods

### *Use of Human Tissues*

Human tissues specimens were approved for use by Institutional Review Boards as follows. All tissues involved by lymphoid neoplasms (save one) were collected and used without informed consent under Brigham and Women’s Hospital IRB protocol 2010-P002736. A non-small cell lung carcinoma microarray was constructed with tissues obtained from patients without consent under Brigham and Women’s Hospital IRB protocol 2006-P001929. A serous ovarian cancer microarray was constructed with tissues obtained from patients without consent under Brigham and Women’s Hospital IRB protocol 2006-P000321. An angiosarcoma microarray was constructed with tissues obtained from patients without informed consent under the University of Texas M. D. Anderson Cancer Center IRB protocol LAB04-0890. In each of these instances, the studies were granted waivers of consent on the following bases: 1) samples were gathered retrospectively from pathology archives and resulted from routine surgical procedures performed for diagnostic purposes; 2) patient identities were anonymized and completely delinked from unique identifiers; and 3) there was no risk to the participants (only anonymized tissues were used). One T-lymphoblastic lymphoma sample collected as part of a trial of the Merck GSI MK-0752 was used with the written informed consent of the affected patient under Dana Farber/Partners Cancer Center protocol 2004-P002170. A breast cancer microarray containing triple negative tumors (negative for estrogen receptor, progesterone receptor, and *HER2* amplification) was constructed with tissues obtained from patients who provided written informed consent under Dana Farber Cancer Institute IRB protocol 93-085.

### *Use of Mice in Xenograft Studies*

REC-1 and KOPT-K1 cell subcutaneous xenograft studies were performed under Dana Farber Cancer Institute IACUC protocol 04-111. HCC1599, HCC1587, and MB-157 cell subcutaneous xenograft studies were performed under a protocol approved by the IACUC at Merck Oncology. Animal work was conducted according to NIH guidelines. All xenograft-bearing animals were monitored daily by veterinary staff for abnormal behaviors/conditions that might require attention to minimize suffering. Animals were sacrificed by CO_2_ asphyxiation per recommendations of the American Veterinary Medical Association. All animals were maintained in CO_2_ for a minimum of 5 minutes following cessation of respiratory motions.

### *Cell lines*

Cell lines were obtained from the American Tissue Culture Collection (REC-1, MAVER-1, HCC1599, HCC1587, and MB-157 cells) or the Leibniz-Institute DSMZ-German Collection of Microorganisms and Cell Lines (KOPT-K1).

### *Test development and validation studies*

These studies relied on: 1) cell lines with mutations in *NOTCH1* that produce either gain or loss of NOTCH1 function, that were grown as xenografts in mice; 2) normal human squamous mucosae and thymus, in which the anatomic distributions of cells with activated NOTCH1 are known; and 3) a primary human T-ALL of known *NOTCH1* genotype (described below). Xenografted cell lines and associated *NOTCH1* genotypes are given in Table 1. All xenografted tumors were fixed in a phosphate buffered solution containing 10% formalin or 4% paraformaldehyde (squamous cell carcinomas) and embedded in paraffin, and were retrieved from the tissue archives of individual laboratories in which xenograft studies were undertaken as part of other studies. Bones were decalcified following formalin fixation using RDO Rapid Decalcification Solution (Sigma, St. Louis, MO).

**Table 1:** Cancer xenografts and associated gain-of-function mutations in NOTCH1.

Cell Line	Tumor Type	NOTCH1 Mutation	Effect on NOTCH1 Signaling	Reference
KOPT-K1	T-LL	L1600P/del(P)*	Gain	[3]
REC-1	MCL	del(ECN1)/del(P)*	Gain	this paper/ [7]
MAVER-1	MCL	None	N.A.	[7]
HCC1599	Breast Carcinoma	del(ECN1)	Gain	[10]
MB-157	Breast Carcinoma	del(ECN1)	Gain	Unpublished data
HCC1587	Breast Carcinoma	None	N.A.	[10]

* X/Y indicates paired mutations created by aberrations in *cis* in the same *NOTCH1* allele del(ECN1) indicates the present of a deletion that removes the coding sequence for the extracellular domain of NOTCH1 del(P) indicates the presence of frameshift or stop codon mutations that remove the C-terminal NOTCH1 PEST degron domain T-LL, T lymphoblastic leukemia/ lymphoma; MCL, mantle cell lymphoma; N.A., not applicable

### *Archival human cancer samples*

All FPE samples were used following approval of institutional review boards (see ethic statements). A primary T-ALL sample known to bear activating mutations in *NOTCH1* was collected as part of a clinical trial of the gamma-secretase inhibitor MK-0754 in patients with relapsed/refractory T-ALL [22]. Archival “discarded” T-LL, CLL, MCL, angiosarcoma, and normal tissue samples were selected from the paraffin-embedded tissues archives of Brigham and Women’s Hospital based solely on the availability of tissue fixed in 10% formalin or in B+, a buffered fixative containing 3.7% formaldehyde and zinc chloride that is widely used for tissues involved by lymphoid neoplasms. FPE non-small cell lung cancer [23], diffuse large B cell lymphoma [24], angiosarcoma [25], and ovarian cancer [26] specimens were studied by staining previously described tissue microarrays. A new tissue microarray containing a set of 78 triple negative breast cancers was assembled as follows. Archival formalin-fixed, paraffin-embedded breast cancers were collected and best blocks and best areas for coring were identified and selected by a breast pathologist (DD). Results of immunohistochemical studies for estrogen (ER) and progesterone receptor (PR) and HER2 and FISH assay results for HER2 were extracted from pathology reports. Seventy-eight triple negative (ER/PR/HER2 negative) invasive breast cancers were identified with adequate tumor tissue for tissue microarray (TMA) construction, which was carried out in the Dana Farber/Harvard Cancer Center Tissue Microarray Core Facility. Three 0.6 mm cores were taken from marked areas and placed into a recipient block using a manual arrayer (Beecher Instruments).

### *Immunohistochemistry*

Standard 4-micron paraffin embedded tissue sections were stained using the Ventana Benchmark XT platform (Ventana Medical Systems, Inc., Tucson, AZ.) with extended heat-induced epitope retrieval (CC1 Buffer). Slides were incubated for 1hr at room temperature with anti-NICD1 rabbit monoclonal antibody (clone D3B8, catalog #4147, Cell Signaling Technology, Beverly, MA; final concentration, 8.5microgram/mL). Signals were then amplified (Ventana Amplification Kit, #760-080) and visualized (Ventana Ultraview Universal DAB detection kit, #760-500) per the manufacturer’s instructions. All staining runs included two positive control samples (REC-1 cell xenograft and tonsillar mucosa). Scoring was performed by two pathologists (MJK, JCA). Staining results were also reviewed with pathologists with subspecialty expertise in hematopathology (MJK, JCA, GP, DD, EAM, EFM), lung pathology (LC), gynecologic pathology (MH), breast pathology (DD), and soft tissue pathology (JH, AL), all of whom also contributed to case selection.

### *NOTCH1* sequencing

DNA was prepared from fresh-frozen samples using the QiaAmp DNA Mini kit (Qiagen, Valencia, CA). Sanger sequencing of *NOTCH1* mutational hotspots in a case of T-lymphoblastic lymphoma was performed as described [3]; sequencing traces with analyzed with Mutation Surveyor Software (Softgenetics, State College, PA). Deep sequencing of *NOTCH1* mutational hotspots was performed as follows. Primers were designed and validated by Fluidigm (Fluidigm Corporation, San Francisco, CA) for exons 25-28 and 34 of *NOTCH1* per recommended guidelines for Roche Titanium sequencing (Roche, Mannheim, Germany). A total of 2304 amplicons representing 48 unique samples amplified with 48 target specific primer pairs were generated in each sequencing run. Each primer included sample-specific Fluidigm 454 bar code primer and adapter sequences. Reactions contained 50ng genomic DNA, forward and reverse tagged amplification primers (1microM), forward and reverse bar code primers (400nM), 1x Access Array Loading Reagent, 1x FastStart High Fidelity Reaction Buffer, 4.5mM MgCl_2_, 5% DMSO, 0.05U FastStart High Fidelity Enzyme Blend and PCR-grade nucleotide mix (200microM, Roche). Thermal cycling was performed on the Fluidigm FC1 Cycler per manufacturer guidelines. The resultant libraries were harvested and collected on a microtiter plate, where they were quantified using the Qubit® dsDNA BR Assay Kit on a Qubit® 2.0 Fluorometer (Invitrogen, UK). Libraries were normalized and pooled before purification using Agencourt AMPure XP system (Beckman, UK) per the manufacturer’s protocol. Library components were clonally amplified using the GS Junior emPCR Lib-A Kit (Roche) by inputting 1 molecule of library DNA per capture bead. Pyrosequencing was performed using the GS Junior system (Roche/454 Life Sciences).

To selectively detect the codon 2514 del(CT) mutation in *NOTCH1*, a pyrosequencing assay was developed. Briefly, *NOTCH1* exon 34 target sequence was amplified in a PCR containing 30ng of genomic DNA, a biotinylated forward primer (5'-CTCCTCGCCTGTGGACAA) and a reverse primer (5'-GGGACGAGCTGGACCACT) using the following cycling parameters: 94°C x 2 minutes, followed 94°C x 30 seconds, 62°C x 30 seconds, 72°C x 30 seconds for 45 cycles, followed by a final extension at 72°C for 10 minutes. The PCR amplification product was captured on streptavidin sepharose beads, denatured, transferred to a pyrosequencing plate, annealed to a reverse sequencing primer (5' CACTGGTCAGGGGACT 3'), and sequenced per a standard protocol (PyroMark Q96 MD, Qiagen, Germantown, MD). The decision to use a reverse sequencing primer was based on the fortuitous presence of a run of 3 G residues immediately 3’ of the ΔG antisense codon 2514 mutation, which acts to increase the sensitivity of the assay roughly 3-fold. In line with this prediction, control studies using the cell line KOPT-K1, a T-LL cell line with a heterozygous del(CT) codon 2514 mutation [3], showed reliable detection of this sequence variant when diluted down to a level of 2.5% mutated alleles with DNA containing wild type *NOTCH1* alleles.

### *Molecular Characterization of an Intragenic NOTCH1 Deletion in REC-1 cells*

5’ rapid amplification of cDNA ends (RACE) was performed on RNA prepared from REC-1 cells and control DND-41 T-ALL cells as described [27]. Genomic DNA was isolated from REC-1 cells using the QIAamp genomic DNA isolation kit (Qiagen, Valencia, CA). Exon1 sense (5’-CCTGCTCTGCCTGGCGCTG) and exon 28 (5’-CCACGAAGAACAGAAGCACA) antisense primers were used to amplify the intragenic Notch1 deletion from 100ng of genomic DNA using the Expand Long Template PCR system (Roche, Branford, CT). The PCR amplification conditions were 94°C x 2 minutes, followed by 94°C x 15 seconds, 60°C x 30 seconds, and 68°C x 45 seconds for 30 cycles, followed by a 68°C extension for 7 minutes. The resulting PCR product was purified, cloned into the pCR2.1-TOPO plasmid (Invitrogen, Carlsbad, CA), and Sanger sequenced.

### *Western Blot Analysis*

Whole cell detergent lysates of cell lines prepared as described [27] were stained for NICD1 (catalog # 4147), total NOTCH1 (catalog #4380), or actin (catalog #4970) using antibodies obtained from Cell Signaling Technologies (Beverly, MA).

### *Xenograft Studies*

REC-1 cells and MAVER-1 cells were inoculated subcutaneously into 6-week old SCID/beige mice (Charles River Laboratory, Wilmington, MA) (1x10^7^ cells per mouse) in 30% Matrigel basement membrane matrix (Becton Dickinson, Franklin Lakes, NJ). Tumor volume was determined by caliper measurements. When tumors reached a volume of 100-200 mm^3^, mice were treated with the gamma-secretase inhibitor diaminobenzidine (DBZ) at 10microM/kg by intraperitoneal injection or with vehicle (DMSO) for 3 days. Tissues were harvested 2hr after the last drug dose. KOPT-K1 cells transduced with a lentivirus that expresses firefly luciferase were injected by tail vein into NOD/SCID/gamma-chain (NSG) mice bred at the Lurie Family Imaging Center (Dana Farber Cancer Institute). Mice were monitored for development of leukemia by bioluminescence and then treated with DBZ or DMSO as above. HCC1587 and HCC1599 human breast cancer cell line xenografts were developed in CB17 mice as described [10], while MB157 cell line xenografts were developed in Swiss nu/nu (nude) mice, all at Merck Oncology. Animal studies were carried out in strict accordance with the recommendations in the Guide for the Care and Use of Laboratory Animals of the National Institutes of Health.

## Results

### *Development of an Immunohistochemical Stain for Activated NOTCH1*

Although canonical Notch signaling relies on nuclear translocation of its intracellular domain (NICD), detection of nuclear NICD *in situ* in human tissues has been difficult. Polyclonal antibodies that detect the neoepitope created at the amino-terminus of NICD1 by cleavage of NOTCH1 at conserved site within its transmembrane domain have been widely used to identify NICD1 in cell lysates, but have not yielded reliable IHC tests. Recent development of a rabbit monoclonal antibody against the NICD1 neoepitope encouraged us to revisit the possibility of using IHC to detect NICD1 in FPE tissues. To limit run-to-run staining variation and to enable possible clinical translation, we evaluated staining on two widely used automated immunostaining platforms, the Ventana Discovery XT and the Leica Bond. Both performed similarly, but we noted that staining interpreted as specific (as per below) appeared stronger on the Ventana platform (data not shown), which was used for all of the studies reported herein.

**Figure 1:**
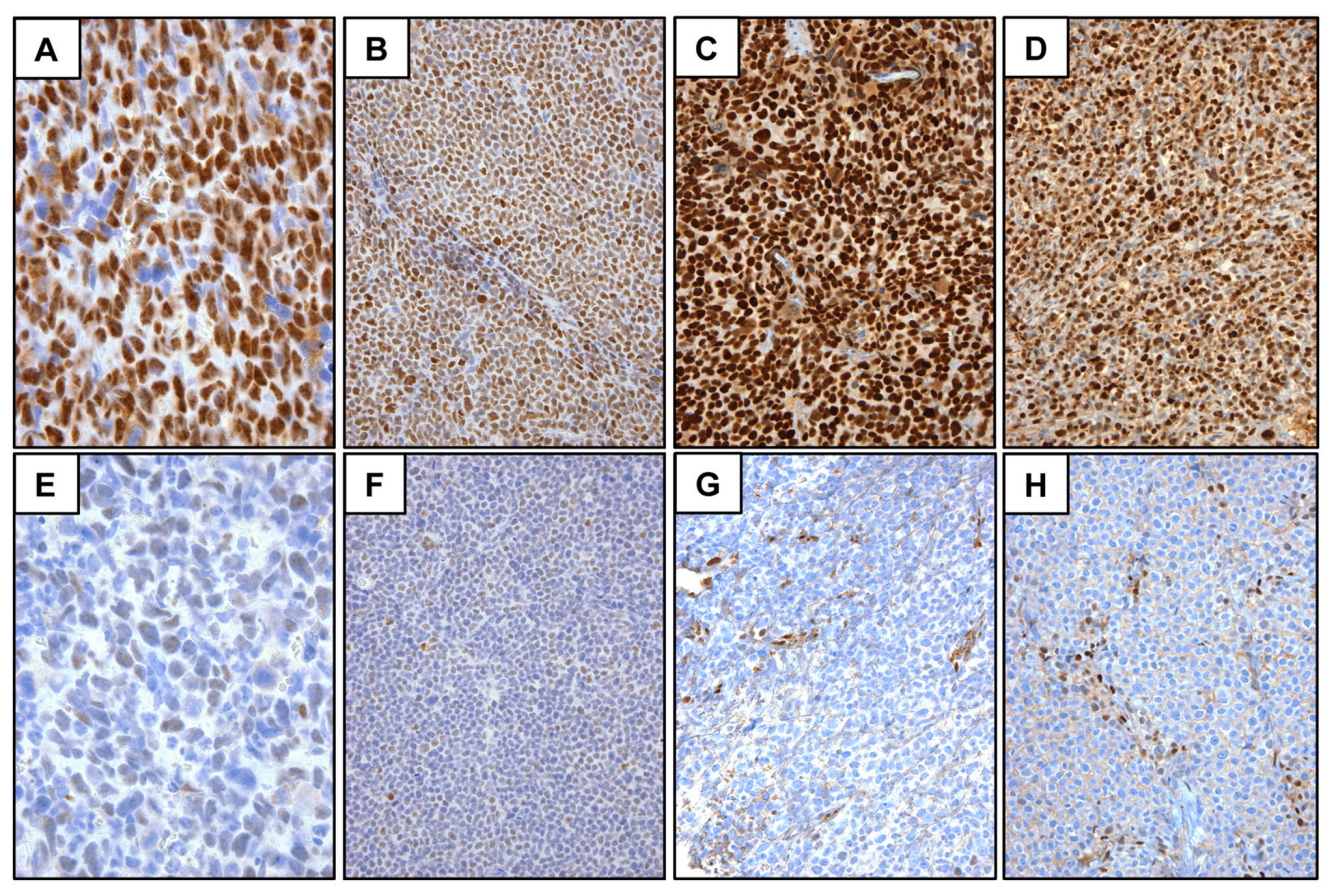
Validation of IHC staining of NICD1 using formalin FPE tumor xenografts. Sections were stained for NICD1 using an IHC method that produces a brown nuclear stain and counterstained with hematoxylin. A) KOPT-K1 T-ALL cells. B) REC-1 mantle cell lymphoma cells. C) HCC1599 breast carcinoma cells. D) MB-157 breast carcinoma cells. E, F) KOPT-K1 and REC-1 cell staining, respectively, in tissues harvested from animals treated with the GSI DBZ. G) MAVER-1 mantle cell lymphoma cells. H) HCC1587 breast cancer cells. Genotypes of these cell lines are given in Table 1.

To develop a reliable staining method for NICD1, we made use of control FPE tissues from mice xenografted with human tumor cell lines bearing oncogenic *NOTCH1* mutations that result in ligand-independent generation of NICD1 (Table 1). Tumors studied with gain-of-function *NOTCH1* mutations included a T-LL cell line, KOPT-K1, which has an activating point substitution in the NOTCH1 negative regulatory region aligned in *cis* with a codon 2514 del(CT) mutation that results in deletion of the C-terminal NOTCH1 PEST degron domain [3]; the MCL cell line REC-1, which bears a *NOTCH1* allele with a previously uncharacterized activating deletion removing most of the coding sequence for the NOTCH1 ectodomain aligned in *cis* with a nonsense mutation in codon 2428 (described below) that also results in deletion of the C-terminal PEST degron domain; and two breast cancer cell lines, HCC1599 and MB-157, which bear *NOTCH1* alleles with deletions that remove most of the NOTCH1 ectodomain coding sequences ( [10]; and data not shown). Formalin FPE sections of all four xenografts bearing activating *NOTCH1* mutations showed strong diffuse nuclear reactivity when stained with the NICD1-specific antibody (Figure 1A–D). Notably, NICD1 staining in KOPT-K1 and REC-1 xenografts was markedly reduced by treatment of animals with the GSI DBZ (Figure 1E, F), which blocks the generation of NICD1 ( [3]). In addition, no NICD1 staining was seen in sections of the MCL cell line MAVER-1 (Figure 1G), which has wild type *NOTCH1* alleles and is devoid of NICD1 [7], or in the breast cancer cell line HCC1587 (Figure 1H), which has wild type *NOTCH1* alleles and instead has an activating deletion involving *NOTCH2* [10].

**Figure 2:**
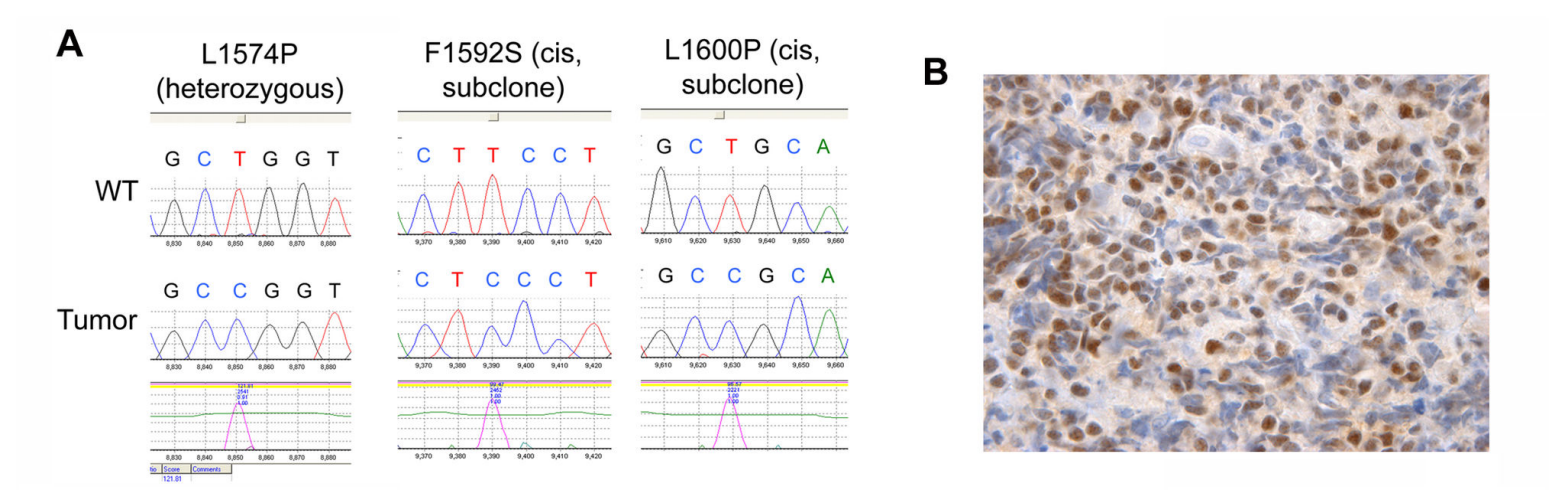
NICD1 staining in an archival human T-LL of known genotype. A) Identification of activating mutations involving exon 26 of *NOTCH1*. Of 24 individual cloned PCR products, 13 gave wild type sequences, 9 showed a mutation producing a L to P substitution in residue 1574, 1 showed the L1574P mutation in *cis* with a mutation producing a F to S substitution in residue 1592, and 1 showed the L1574P mutation in *cis* with a variant producing a L to P substitution in residue 1600. B) IHC staining for NICD1 in the same tumor, a formalin-fixed paraffin-embedded biopsy of a mediastinal mass. There is crush artifact, but strong nuclear staining is seen in intact cells.

To further evaluate the sensitivity of the method and the stability of the NICD1 neoepitope in archival tissues, we performed IHC for NICD1 on sections prepared from a T-LL specimen obtained in the year 2004 from a patient enrolled in a trial of the Merck GSI MK-0754 [22]. Sanger sequencing of DNA isolated from this specimen revealed a heterozygous point mutation that results in a L1574P substitution, as well as two additional subclonal point mutations aligned in *cis* with the L1574P substitution that create L1600P and F1592S substitutions (Figure 2A). All of these mutations fall in exon 26 of *NOTCH1* and correspond to previously characterized gain-of-function mutations in the NOTCH1 negative regulatory region [28], a portion of the NOTCH1 ectodomain that must be intact to keep the receptor in the off-state in the absence of ligand [29]. Tandem mutations aligned in *cis* in the NOTCH1 ectodomain have been reported previously [3] and are likely a manifestation of continued selection for increased NOTCH1 signaling during T-ALL progression. IHC staining of this T-ALL produced strong diffuse nuclear positivity, suggesting that the NICD1 epitope detected by the rabbit monoclonal antibody is stable for a period of years in routinely stored FPE samples (Figure 2B).

**Figure 3:**
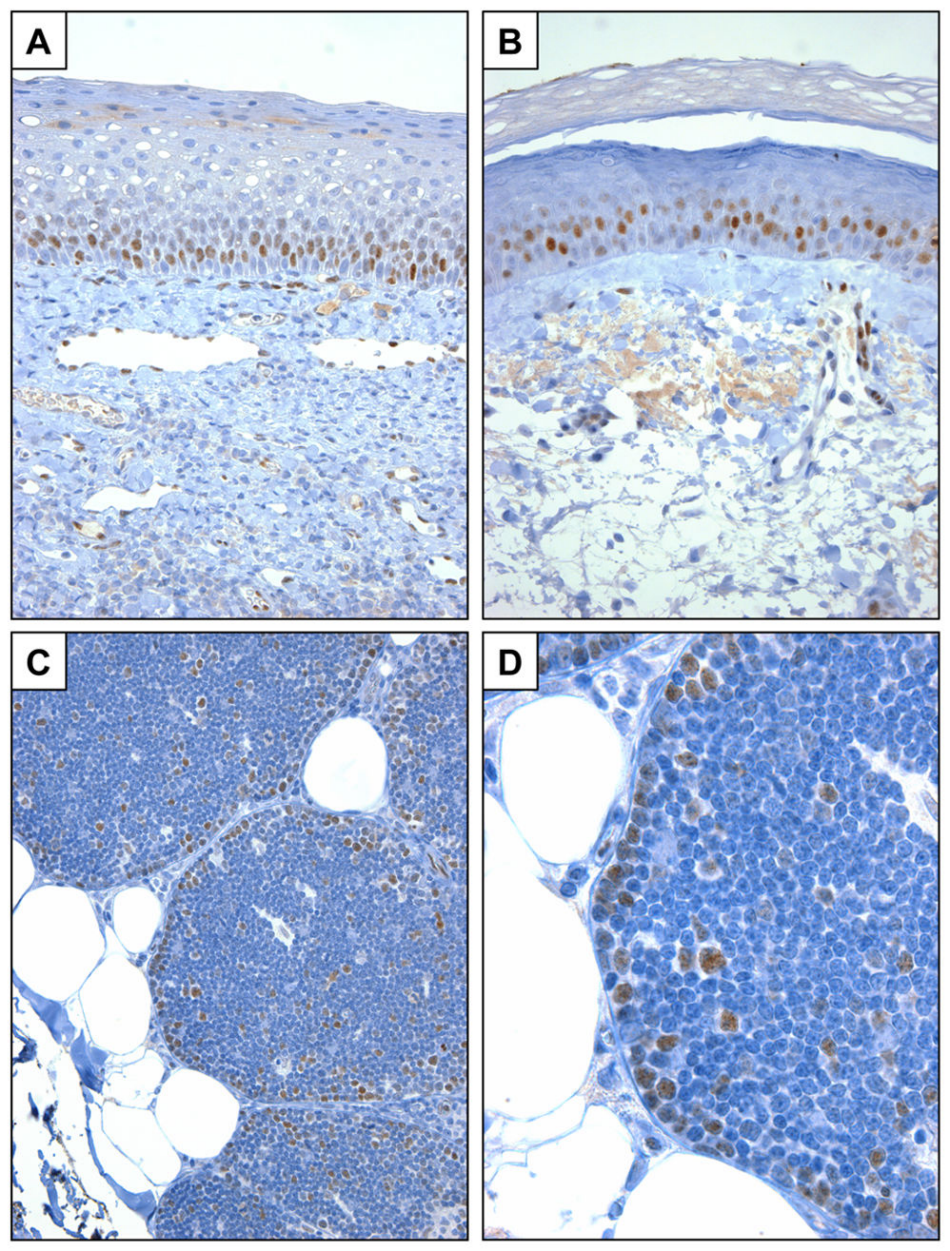
NICD1 staining in normal formalin FPE human tissues. A) Oropharyngeal squamous mucosa. B) Skin. C, D) Thymus.

We also noted in sections of xenografted tumors that murine endothelial cells and scattered stromal cells showed nuclear reactivity with the NICD1 antibody (best seen in Figure 1G and H). These observations are in line with data indicating roles for notch1 in vascular endothelium [30] and peripheral T cells [31], and suggested that our IHC method can detect physiologic levels of NICD1. To evaluate this, we stained normal sections of human squamous mucosa, skin, and thymus (Figure 3). Prior work has shown that notch1 is transiently activated in suprabasilar cells in squamous epithelia [32], in which it promotes cell cycle exit and differentiation [33], an activity that may contribute to NOTCH1’s tumor suppressive roles in squamous cell carcinomas of the skin and head and neck. As expected, NICD1 staining in squamous epithelia was confined to suprabasilar cells (Figure 3A, B). In thymocytes, notch1 activation rises to a maximum in CD4/CD8 double-negative cells undergoing beta-selection [34], which are located within the thymic cortex just deep to the thymic capsule [35]. In prior work, we noted that cells in this region of the thymus have the highest level of immunoreactivity with antibodies that recognize total notch1, without regard to its activation status [36]. As predicted from the reported pattern of NOTCH1 activation in thymocytes, NICD1 staining in human thymus was most prominent in cortical thymocytes lying immediately below the capsule (Figure 3C, D). These results demonstrate that our IHC method is sufficiently sensitive to detect physiologic levels of activated NOTCH1 in at least some normal tissues. To further explore NOTCH1 activation in normal lymphoid tissues, staining of tonsil was also carried out. We noted that germinal centers containing reactive B cells were devoid of immunoreactivity, whereas surrounding mantle zones showed weak staining for NICD1 (data not shown).

### *Detection of NOTCH1 Activation in Archival Cancer Specimens*

Building on these validation studies, we next used IHC to screen a series of FPE human cancers for evidence of NOTCH1 activation, focusing on tumor types in which *NOTCH1* gain-of-function mutations have been described. We also studied angiosarcoma, based on the observation that normal endothelial cells frequently stained positively for NICD1 and prior studies suggesting that *notch1* has a tumor suppressive function in murine vascular endothelium [19,20]; hence, we were curious to see if NOTCH1 activation might be lost in human angiosarcomas. We used NICD1 staining of normal endothelial cells as an internal control to judge the preservation of immunoreactivity in the tissues studied. In exploratory studies, we noted that archival samples fixed in Zenker’s solution, a mercuric fixative used at our institution to decalcify bone marrow biopsies, negated NICD1 immunoreactivity in endothelium. Loss of immunoreactivity was not caused by decalcification *per se*, since bone marrow involved by xenografted KOPT-K1 cells that was fixed in formalin and then rapidly decalcified with a strong acid retained immunoreactivity (Figure 1A). Because of this limitation, we confined our studies to tumors involving soft tissues for which FPE samples were available. Some lymph nodes involved by CLL samples were fixed in B+, a fixative that contains formaldehyde and zinc chloride. Samples fixed in B+ retained NICD1 staining in normal endothelial cells and thus were included in the analysis. Results are summarized in Table 2 and are described in the sections below.

**Table 2:** Summary of NICD1 staining in human FPE cancer specimens.

Tumor (Number of Cases)	NICD1 Staining Results
T-LL (N=14)	57% positive (N=8; all diffusely positive)
CLL (N=52)	89% positive (N=46; 15% diffusely positive, 74% subset positive)
Peripheral T cell lymphoma (N=55)	38% positive (N=21; 1.8% diffusely positive, 36% subset positive)
Mantle cell lymphoma (N=53)	0% positive
Diffuse large B cell lymphoma (N=68)	0% positive
Triple negative breast carcinoma (N=78)	3.8% positive (N=3; all diffusely positive)
Non-small cell lung carcinoma (N=151)	0% positive
Ovarian carcinoma (N=147)	0% positive
Angiosarcoma (N=57)	77% positive (N=44; 47% diffusely positive, 30% subset positive)

Diffusely positive, ≥80% of cells positive; subset positive, ≤80% of cells positive

### *T lymphoblastic leukemia/lymphoma *(*T-LL*)

**Figure 4:**
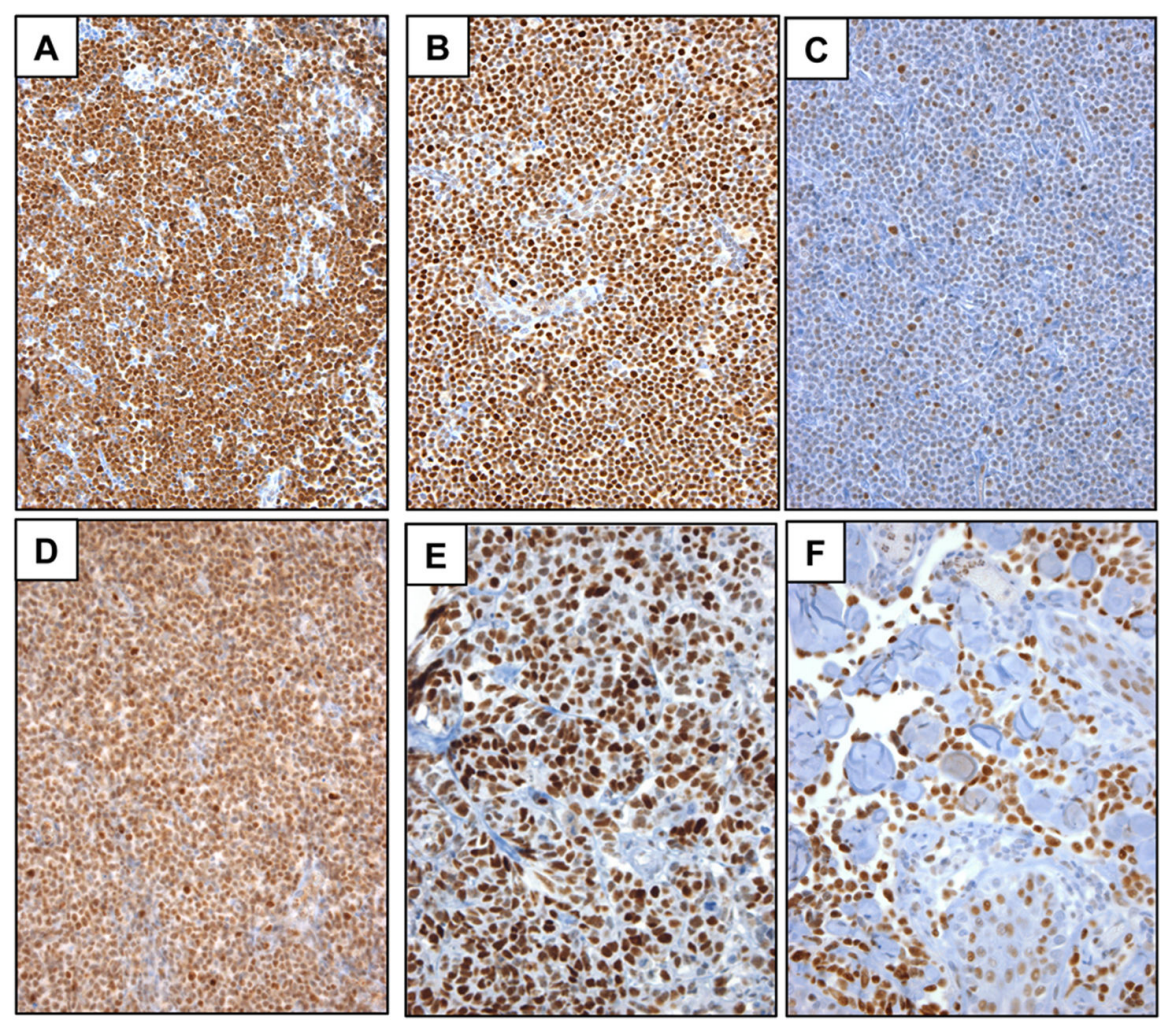
NICD1 immunoreactivity in archival FPE human cancers. A) T-ALL. B) CLL, diffuse pattern. C) CLL, subset pattern. D) Adult T-cell leukemia/lymphoma. E) Triple-negative breast carcinoma. F) Angiosarcoma.

*NOTCH1* gain-of-function mutations occur in roughly 50% to 60% of human T-LL [3] and a high fraction of murine T-LL models. Consistent with the known incidence of *NOTCH1* gain-of-function mutations in human disease, 8 of 14 T-ALLs screened (57%) showed diffuse nuclear staining for NICD1 (Figure 4A). In tumors that were negative, little or no staining was observed. This black and white distinction—diffuse positivity or no staining—is consistent with the idea that T-LL falls into two major classes, one in which NOTCH1 is “on” by virtue of acquired gain-of-function mutations and a second that is unrelated to NOTCH1 activation and free of *NOTCH1* mutations.

### *Mantle cell lymphoma *(*MCL*)

A recent report identified *NOTCH1* exon 34 mutations in 11% of MCL and in the MCL cell line REC-1, while certain other MCL cell lines such as MAVER1 were free of NOTCH1 mutations [7]. The high levels of NICD1 in REC-1 cell line extracts [7] and the intense immunoreactivity of xenografted REC-1 cells stained for NICD1 suggested that this cell line might harbor additional mutations leading to ligand-independent activation of NOTCH1, since exon 34 mutations affecting the NOTCH1 PEST domain have no effect on NICD1 generation *per se* [3,28]. The most common mutations leading to ligand-independent activation of NOTCH1 in human T-LL are point mutations in the extracellular negative regulatory region, but sequencing of REC-1 failed to identify mutations in this region [7]. We previously noted that murine T-LLs often have activating deletions that remove the coding sequences for the notch1 extracellular negative regulatory region [27], and similar lesions in human NOTCH1 occur in a subset of human breast cancers and breast cancer cell lines such as HCC1599 and MB-157 [10].

**Figure 5:**
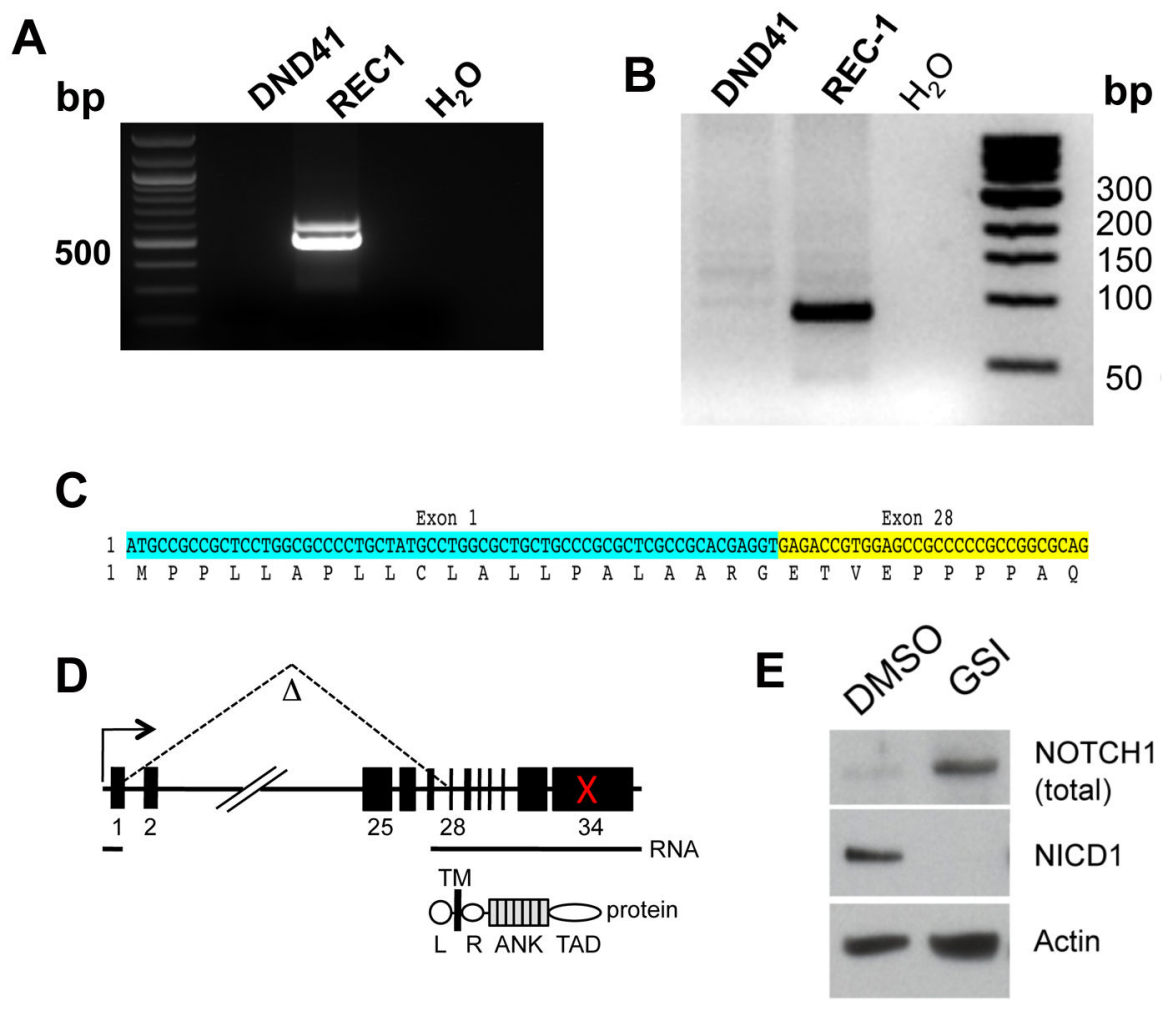
Identification of an activating deletion in *NOTCH1* in REC-1 mantle cell lymphoma cells. A) 5’-RACE products synthesized from input RNA from REC-1 MCL cells and DND-41 T-ALL cells; the latter have intact *NOTCH1* alleles. B) Results of PCR of genomic REC-1 and DND-41 cell DNA with a *NOTCH1* exon 1/exon 28-specific primer pair. C) Sequence of the 5’-RACE product in A and the genomic PCR product in B. Both showed an in-frame fusion of exon 1 and exon 28 *NOTCH1* coding sequences. D) Cartoon depicting the intragenic *NOTCH1* deletion in REC-1 cells and its consequences at the level of NOTCH1 RNA and protein. The red X denotes the position of a frame-shift mutation in exon 34 of *NOTCH1*. L, leader peptide; TM, transmembrane domain; R, RAM domain; ANK, ankyrin repeat domain; TAD, transcriptional activation domain. E) Western blot showing the effect of treatment of REC-1 cells with the gamma-secretase inhibitor (GSI) compound E for 72 hours versus DMSO control. GSI depletes NICD1 (detected with the NICD1-specific V1744 antibody) and leads to the accumulation of a polypeptide of the size predicted by virtual translation of the aberrant *NOTCH1* mRNA. Total NOTCH1 was detected with a polyclonal antibody raised against the NOTCH1 TAD.

We thus asked whether REC-1 cells might harbor a *NOTCH1* deletion leading to expression of an aberrant *NOTCH1* mRNA. Using 5’-rapid amplification of cDNA ends (5’-RACE), we identified an abnormal transcript consisting of an in-frame fusion of *NOTCH1* exon 1 and exon 28 sequences (Figure 5A, C). PCR amplification of genomic REC-1 DNA produced a product with the same sequence (Figure 5B, C), consistent with the presence of a *NOTCH1* deletion created by DNA breaks in exon 1 and exon 28 followed by non-homologous end joining. Virtual translation of the aberrant REC-1 transcript produces a membrane-tethered form of NOTCH1 closely resembling truncated forms of NOTCH1 that are potent leukemogens in mice [37] and that undergo ligand-independent cleavage by gamma-scretase to yield NICD1 [38]. In line with this prediction, treatment of REC-1 cells with GSI led to accumulation of a truncated NOTCH1 protein and disappearance of NICD1 (Figure 5D).

Based on the findings in REC-1 cells and the report of *NOTCH1* mutations in 11% of MCLs [7], we anticipated that NICD1 staining would be observed in a significant subset of MCLs. However, IHC staining in 53 cases of MCL was uniformly negative, suggesting that NOTCH1 activation and (by extension, *NOTCH1* mutations) were absent from our cohort of cases, which were diagnosed by experienced hematopathologists using standard World Health Organization criteria. In line with the IHC results, deep sequencing of DNAs prepared from all MCLs for which frozen tissue was available (N=45) failed to detect any mutations in *NOTCH1* exons encoding the extracellular negative regulatory region (exons 25-27), the gamma-secretase cleavage site (exon 28), or the C-terminal PEST degron domain (exon 34) (data not shown). Based on prior structure: function studies [28,39], these exons span all regions of *NOTCH1* that are predicted to potentially harbor gain-of-function point mutations and small insertions and deletions. Thus, although activating *NOTCH1* mutations clearly occur in MCL, they may be less common than originally thought.

### *Chronic lymphocytic leukemia *(*CLL*)

In contrast to T-LL and MCL, staining for NICD1 in CLL was both more frequent and more variable. In total, 89% of CLLs (46 of 52 cases) stained positively for NICD1 in >10% of tumor cells; of these 33% (17 of 52) stained positively in >50% of tumor cells, and 15% (8 of 52) showed diffusely positive staining (defined as staining in >80% of cells). Examples of diffusely positive cases and subset-positive cases are shown in Figure 4B and 4C, respectively. From 10% to 18% of CLLs have been reported to have *NOTCH1* gain-of-function mutations in prior studies [4–6]; thus, the frequency of NOTCH1 activation as judged by NICD1 staining in our tumor cohort greatly exceeds what would be expected if activation were confined to CLLs with *NOTCH1* mutations.

**Figure 6:**
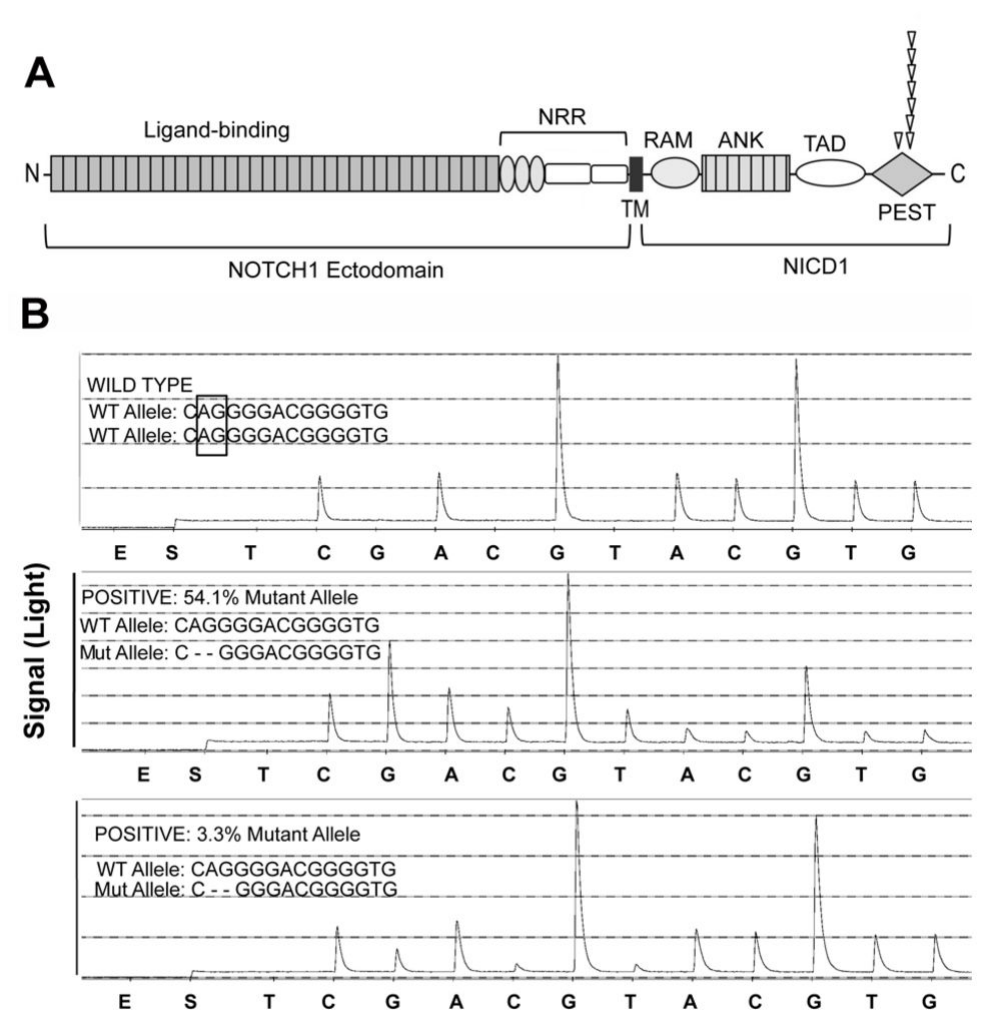
*NOTCH1* mutations in CLL. A) Position of *NOTCH1* mutations, superimposed on a cartoon showing the NOTCH1 protein. NRR, negative regulatory region; RAM, RAM domain; ANK, ankyrin repeat domain; TAD, transcriptional activation domain; PEST, degron domain. B) Representative confirmatory pyrosequencing results in CLL cases positive by deep sequencing for the *NOTCH1* codon 2514 del(CT) mutation. Results for a case with wild type codon 2514 sequences is shown in the upper panel. The lower two panels are cases in which deep sequencing revealed high (54.1%) or low numbers of mutated reads (3.3%). Reverse-sequences of the wild type (WT) and mutant (MUT) del(CT) alleles are shown for reference. “E” corresponds to the addition of enzyme to the reaction chamber, while “S” corresponds to the addition of the substrate. In this sequencing by synthesis assay, nucleotides are added to the reaction chamber sequentially in the order shown. Nucleotide incorporation releases pyrophosphate, which catalyses a reaction in which the number of photons produced is proportional to the number of nucleotides incorporated. The codon 2514 del(CT) mutation causes the appearance of a new signal corresponding to the incorporation of 3 G residues.

To evaluate genotype/phenotype relationships directly, deep sequencing was performed on all CLL samples evaluated by NICD1 staining for which fresh-frozen tissue was available (N=46). Fold coverage of reads in most samples was >100, sufficient to allow detection of minor subclones. We detected *NOTCH1* gain-of-function mutations in 8 of 46 cases (17%), all in exon 34. Seven corresponded to a del(CT) mutation in codon 2514 that is highly prevalent in CLL, and one corresponded to a nonsense mutation in codon 2444 (Figure 6A); mutations at both of these sites result in disruption of the C-terminal PEST degron domain. Six mutations were present in dominant clones (defined as >20% mutated reads), whereas two mutations were present in minor subclones (defined as <5% mutated reads). All seven codon 2514 del(CT) mutations were confirmed using a pyrosequencing assay capable of detecting approximately 2.5% mutated sequences diluted into wild type DNA; representative pyrosequencing results are shown in Figure 6B. The *NOTCH1* codon 2444 nonsense mutation was confirmed by repeat deep sequencing of DNA from two additional frozen pieces of tissue from this case. In the first piece of tissue analyzed, the codon 2444 mutation was present in 46% of reads, and in the two additional pieces the same mutation was present in 33% and 48% of the reads (data not shown), confirming that this mutation is also real.

**Figure 7:**
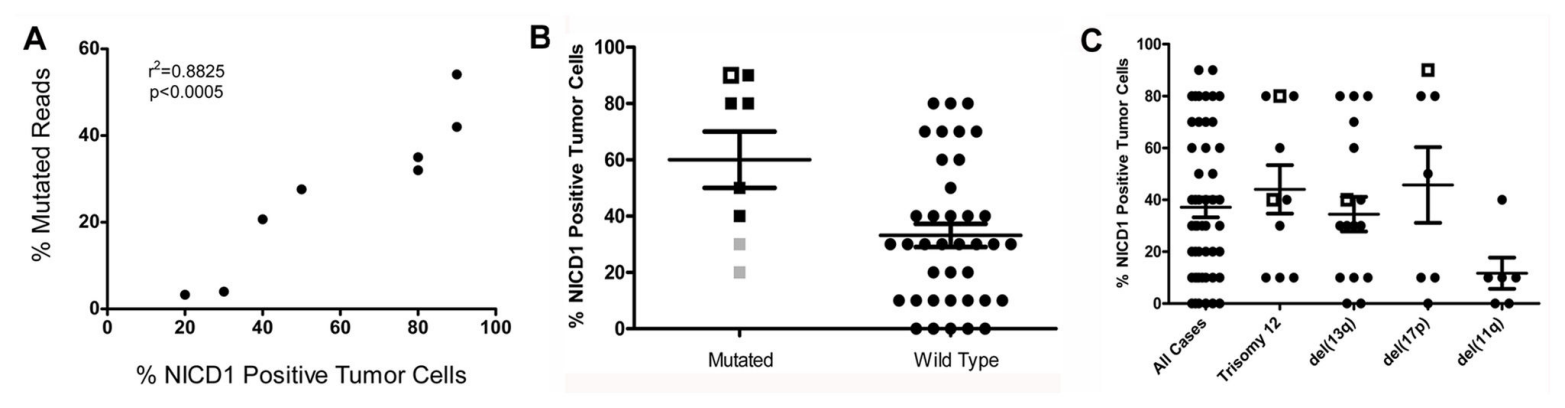
Relationship of NICD1 staining, *NOTCH1* genotype, and karyotype in CLL. A) Correlation between the fraction of mutated *NOTCH1* exon 34 reads and NICD1 staining. B) NICD1 staining in CLLs with and without mutated *NOTCH1* alleles. Filled squares correspond to CLLs with the NOTCH1 codon 2514 del(CT) mutation; black squares are tumors with a “high” fraction (>20%) of mutated reads, while gray squares are tumors with a “low” fraction (<5%) of mutated reads. The open square corresponds to a CLL with a nonsense mutation in codon 2444. C) Relationship of NICD1 staining to CLL karyotype. Open squares correspond to tumors with *NOTCH1* mutations.

We then correlated NOTCH1 activation with *NOTCH1* genotype. The fraction of reads with *NOTCH1* mutations in a particular sample was strongly correlated with NICD1 staining (Figure 7A), in line with the expectation that PEST deletions should stabilize NICD1 and increase its levels. Cases with *NOTCH1* mutations had significantly more widespread staining for NICD1 than cases with wild type alleles (Figure 7B, p<0.05); however, there was substantial overlap between these two groups of tumors, and some tumors with wild type *NOTCH1* alleles were diffusely positive for NICD1 (>80% of cells positive). We also assessed the relationship between NOTCH1 activation and cytogenetic abnormalities, which are widely used markers of prognosis in CLL. Prior work showed enrichment for *NOTCH1* mutations in CLLs associated with trisomy 12 [40,41], suggesting that this group of tumors might have higher levels of NOTCH1 activation. Among CLLs for which cytogenetic results were available, however, we did not observe any significant difference in NICD1 staining between tumors with trisomy 12, del(13q), or del(17p) (Figure 7C). The most notable observation was low NICD1 positivity in tumors associated with del(11q) relative to other cytogenetics subtypes (p<0.02).

**Figure 8:**
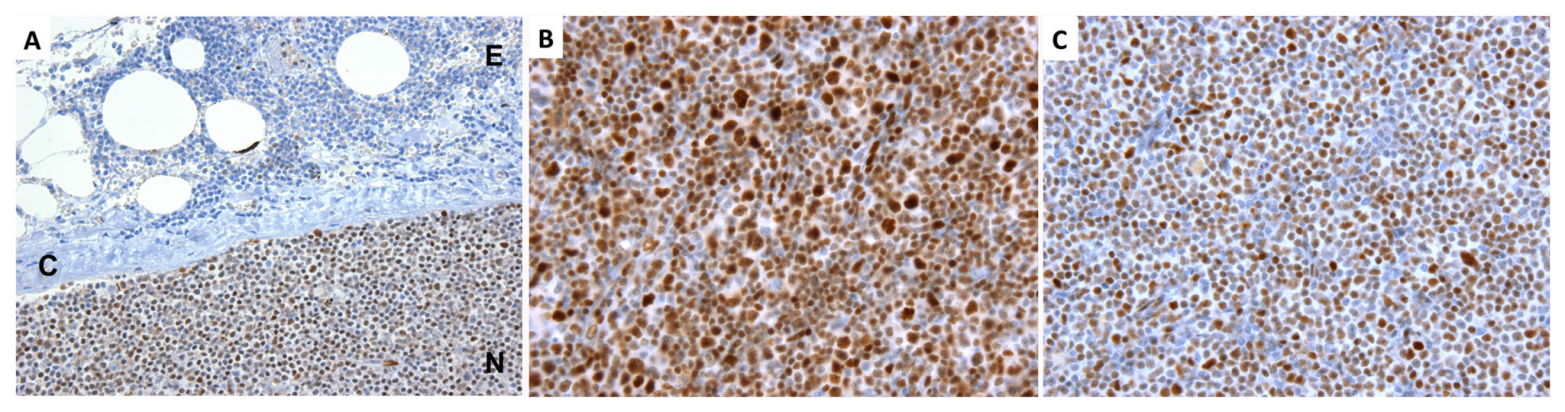
Salient observations from archival CLL biopsies stained for NICD1. A) Loss of NICD1 positivity in extranodal tumor. This case was associated with a *NOTCH1* codon 2514 del(CT) mutation (35% of sequencing reads). E, extranodal fibrofatty tissue; C, lymph node capsule; N, lymph node. B, C) NICD1 staining in sequential biopsies involved by CLL with (B) and without (C) the *NOTCH1* codon 2444 nonsense mutation. The specimen in (B) was associated with 42% mutated sequencing reads; no mutated reads were detected in the subsequent biopsy shown in (C).

Frequent activation of NOTCH1 in CLLs with wild type *NOTCH1* alleles suggests that microenvironmental factors (e.g., Notch ligands) mediate NOTCH1 activation in this disease. Little is known about Notch ligand expression in lymph nodes, and reliable IHC staining methods for the full set of Notch ligands (Jagged1, Jagged2, Delta-like-1, Delta-like-3, and Delta-like-4) have yet to be developed, limiting our ability to directly assess the possible involvement of ligands. However, several “experiments of nature” in our tumor set point to a role for intranodal factors in NOTCH1 activation. In 13 of the CLL cases studied, tumor cells extended beyond the lymph node capsule into perinodal soft tissue. In 12 of these 13 cases, we noted that NICD1 staining was much weaker in tumor cells in perinodal soft tissue than it was within tumor cells in the immediately adjacent lymph node, including one case associated with a the *NOTCH1* codon 2514 del(CT) in 35% of sequencing reads (Figure 8A). In addition, the case with a NOTCH1 codon 2444 nonsense mutation in 42% of sequencing reads subsequently recurred as a tumor with wild type *NOTCH1* alleles (Figure 8B, C). The original mutated sample showed evidence of transformation to a more aggressive histologic grade as judged by the presence of frequent large cells, a feature associated with *NOTCH1* mutations in a prior report [5], whereas the later lymph node biopsy showed only a few scattered large cells. NICD1 staining was somewhat stronger in the earlier tumor with mutated *NOTCH1*, but both biopsies showed diffusely positive NICD1 staining. These observations are consistent with past work showing that exon 34 mutations producing C-terminal PEST domain deletions have no effect on receptor activation *per se* [3,28]; thus, they are only selected for in contexts in which there is already ongoing NOTCH1 activation, which may be caused by mutations in exons 25-28 (apparently absent in CLL) or by ligand-mediated NOTCH1 activation.

### *Diffuse large B cell lymphoma*

Mutations in *NOTCH1* have been reported in a minor subset of diffuse large B cell lymphoma [8]. However, staining of a tissue microarray containing 68 well-characterized diffuse large B cell lymphomas revealed no NICD1 positive tumors (Table 2). Thus, NOTCH1 activation is uncommon in diffuse large B cell lymphoma.

### *Adult T-cell leukemia/lymphoma and other peripheral T cell lymphomas*

**Figure 9:**
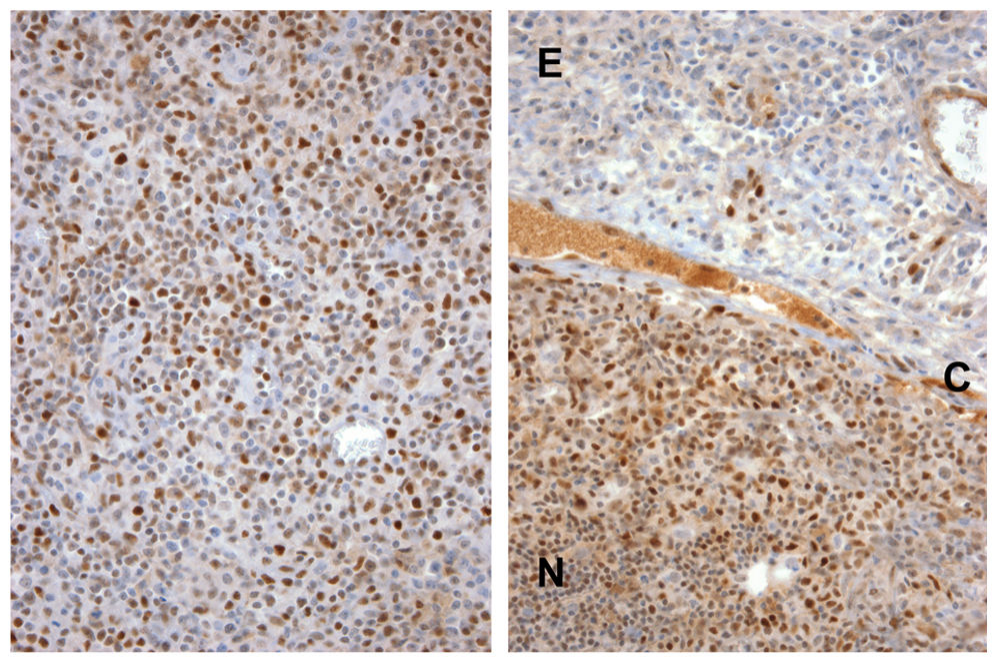
Representative NICD1 staining in angioimmunoblastic T cell lymphoma. Left, intranodal tissue, showing variable staining of resident cells. Right, micrograph showing a sharp decrease in NICD1 staining in extranodal (E) tumor cells as compared to nodal (N) tumor cells, which are separated by the nodal capsule (C).

Staining of a diverse collection of peripheral T cell lymphomas, subclassified by World Health Organization criteria, revealed NICD1 positivity in 21 of 55 cases (38%; Table 3), including diffuse nuclear positivity in 1 case of adult T cell leukemia/lymphoma (Figure 4D). Also noted was a variable pattern of NICD1 immunoreactivity in 12 of 14 cases (86%) of angioimmunoblastic lymphoma, a T cell lymphoma derived from follicular T helper cells (Figure 9). As with CLL, NICD1 staining was diminished in angioimmunoblastic lymphoma cells in extranodal soft tissues, suggesting that NICD1 production is also stimulated in this tumor-type by factors in the nodal microenvironment (Figure 9).

**Table 3:** NICD1 staining in peripheral T cell lymphomas.

Tumor Type (N=55 Total Cases)		NICD1 Staining Results	
Angioimmunoblastic lymphoma (N=14)		86% positive (N=12; all subset positive)	
Peripheral T cell lymphoma, NOS (N=12)		42% positive (N=5; all subset positive)	
Cutaneous T cell lymphoma (N=12)		17% positive (N=2; all subset positive)	
Anaplastic large cell lymphoma (ALCL, N=7)		14% positive (N=1; subset positive, ALK+ tumor)	
		86% negative (N=6, 1 ALK+ tumor, 5 ALK-tumors)	
Adult T cell leukemia/lymphoma (N=5)		20% positive (N=1; diffusely positive)	
EBV+ T/NK extranodal lymphoma (N=4)		0% positive	
Subcutaneous paniculitis-like T cell lymphoma (N=1)		0% positive	

NOS, not otherwise specified; ALK, anaplastic lymphoma kinase; EBV, Epstein–Barr virus; NK, natural killer cell

Diffusely positive, ≥80% of cells positive; subset positive, ≤80% of cells positive

### *Triple-negative breast cancer*

Activating deletions in either *NOTCH1* or *NOTCH2* have been identified in a minority of breast cancers, most of which are triple-negative (ER/PR/HER2 negative) tumors [10]. Staining of a tissue microarray containing 78 triple negative breast cancers for NICD1 identified 3 tumors (3.8%) with strong diffuse nuclear staining (Figure 4E), a pattern similar to that in xenografted breast cancer cell lines with activating deletions of *NOTCH1* (Figure 1C and D).

### *Non-small cell lung cancer *(*NSCLC*)* and Ovarian Carcinoma*

None of 151 NSCLCs or 147 ovarian serous carcinomas had detectable NICD1 staining (Table 2). All cases scored demonstrated staining for NICD1 in vascular endothelium, indicating that lack of staining in tumor cells was unlikely to have resulted from loss of tissue immunoreactivity. Thus, despite reports of NOTCH1 PEST domain mutations in lung cancer [11] and *NOTCH3* amplification and acquired mutations in genes encoding other NOTCH pathway signaling components in ovarian cancer [12,42], NOTCH1 activation does not appear to be common in either of these tumors.

### *Angiosarcoma*

An initial screen of 3 well-differentiated angiosarcomas of the scalp revealed diffuse NICD1 positivity in all 3 tumors (Figure 4F), prompting us to study NOTCH1 activation in an arrayed collection of 54 additional angiosarcomas. Overall, these studies revealed diffuse or focal positive staining for NICD1 in 77% (44 of 57) of angiosarcomas (Table 2). In general, well-differentiated vasoformative tumors showed more diffuse and stronger staining than tumors with epithelioid morphologies or solid growth patterns, both of which are associated with more aggressive behavior.

## Discussion

The method described here for detecting NICD1 in FPE tissues should find wide utility in evaluating NOTCH1 activation in clinical samples and experimental models of human disease. It relies on a commercial rabbit monoclonal antibody and can be performed using standardized reagents on widely used automated staining platforms. The antibody detects a neoepitope at the amino terminus of NICD1 that is created by gamma-secretase cleavage of human NOTCH1 and murine notch1, and which appears to be stable in FPE samples stored routinely for a decade or more. We anticipate that this IHC stain should be useful in a number of contexts, including: i) studies using tissue archives to explore the role of NOTCH1 in various neoplastic and non-neoplastic disorders; ii) selection of patients for trials of gamma-secretase inhibitors (GSIs) [21], the lead Notch pathway antagonists being tested in cancer patients, particularly trials involving diseases such as triple-negative breast cancer in which NOTCH1 activation is confined to a small subset of tumors; and iii) judging the efficacy of NOTCH1 antagonists in experimental models and in patients. Staining for NICD1 may also prove useful in identifying tumors with *NOTCH1* loss-of-function mutations, which are present in a subset of squamous cell carcinomas.

The IHC test that we described has certain limitations. Decalcification of bone marrow with some agents (e.g., Zenker’s solution) seems to negate immunoreactivity, based on staining of T-ALLs for which both soft tissue samples and involved bone marrow were available (data not shown). In addition, the assay is specific for NOTCH1, and therefore cannot be used to assess activation of other NOTCH receptors, which have been implicated in certain cancers. Specifically, activating *NOTCH2* mutations have been identified in marginal zone B-cell lymphoma [43–45] and diffuse large B-cell lymphoma [8], and biochemical analyses have detected activation of NOTCH2 as well as NOTCH1 in CLL [46,47]. Similarly, amplification of *NOTCH3* has been described in ovarian cancers [42,48]. It may be possible to overcome this limitation by developing IHC stains for activated NOTCH2-4, which is feasible in principle since the neoepitopes created by gamma-secretase cleavage of NOTCH1-4 are unique. Finally, a role for NOTCH1 in maintenance of cancer stem cells has been proposed in some tumors [49–52]; if such cells are rare, assessment of NICD1 in bulk tumor cell populations may underestimate the contribution of NOTCH1 to the malignant phenotype.

In addition to a small subset of breast cancers and (as expected) more than half of T-LLs, our screen of human cancers indicates that NOTCH1 activation is prevalent in CLL and also occurs in a subset of peripheral T cell lymphomas and a high fraction of angiosarcomas. An unexpected finding, based on prior results of genomic sequencing studies, was the much more widespread activation of NOTCH1 in CLL than in other two other B cell tumors, mantle cell lymphoma [7] and diffuse large B cell lymphoma [8]. NOTCH1 activation in CLL is not confined to tumors with *NOTCH1* mutations and appears to be augmented by factors expressed in the nodal microenvironment. These findings raise questions about how best to gauge and target NOTCH1 activation in CLL patients who are being considered for treatment with Notch pathway inhibitors. It would be desirable to be able to stratify patients based on analysis of circulating CLL cells, but our findings raise several questions about analyses conducted on peripheral blood. On the one hand, the loss of NICD1 immunoreactivity in CLL cells in soft tissues adjacent to lymph nodes suggests that the levels of NOTCH1 activation differ in CLL cells in different microenvironments within an individual patient. It will be of interest to compare levels of NOTCH1 activation in lymph nodes and peripheral blood cells obtained from patients at the same time point. On the other hand, it appears that *NOTCH1* mutational analysis may underestimate the involvement of NOTCH1 in CLL, based on the presence of readily detectable NOTCH1 activation in the nodal microenvironment in cases with wild type *NOTCH1* alleles. The latter observations suggest that NOTCH1 activation in CLL may be largely ligand-mediated; if true, this would have significant therapeutic implications, since ligand-specific blocking antibodies with antitumor activity have been developed [53,54]. Our observations point to the need for further work on nodal factors that trigger NOTCH1 activation in CLL cells and potentially in other tumors as well. By contrast, while our identification of a novel activating *NOTCH1* deletion in the mantle cell lymphoma cell line REC-1 and prior identification of *NOTCH1* mutations in primary cases of mantle cell lymphoma [7] clearly indicate an oncogenic role for NOTCH1, our failure to detect NOTCH1 activation or *NOTCH1* mutations in 53 well characterized mantle cell lymphomas suggests that involvement of NOTCH1 in this disease is rare.

NOTCH1 has an important role in regulating the differentiation and function of diverse peripheral T cell subsets [55], but its role in peripheral T cell lymphomas is largely unknown. Detection of NICD1 in a substantial subset of peripheral T cell lymphomas suggest that Notch antagonists merit study as potential treatments for these tumors, which have poor outcomes with current therapies.

NOTCH1 is a double-edged sword with respect to tumorigenesis, being capable of serving as a tumor suppressor as well as an oncoprotein. Indeed, inhibition of notch signaling in rodents with inhibitory antibodies directed against the Notch ligand DLL4 leads to tumors in several organs that may be of vascular origin [56], and conditional knockout of *notch1* in endothelial cells causes the appearance of vascular proliferations in the liver [19,20], suggesting that NOTCH1 is a tumor suppressor in endothelium. However, we observed ongoing NOTCH1 activation in a high fraction of human angiosarcomas, particularly in those that are well differentiated. Thus, while loss of NOTCH1 function may be associated with progression of angiosarcoma, it may not be essential for its genesis. These findings suggest that the role of NOTCH1 in transformed endothelium cells merits additional study.

In addition to cancer cells, we observed widespread activation of NOTCH1 in normal endothelial cells and variable activation in mononuclear cells that may represent T cells, macrophages or dendritic cells. Given that NOTCH1 signaling has been implicated in a number of non-neoplastic immunologic, inflammatory and vascular conditions [14,57], the method described here should find widespread use in studying NOTCH1 activation in a broad range of human disorders and corresponding experimental models.
